# An exploratory study on material deprivation and loneliness among older adults in Hong Kong

**DOI:** 10.1186/s12877-024-05013-1

**Published:** 2024-05-06

**Authors:** Vivien Foong Yee Tang, Kee Lee Chou

**Affiliations:** The Education University of Hong Kong, 10 Lo Ping Rd, Tai Po, Hong Kong

**Keywords:** Loneliness, Material deprivation, Poverty, Social resources, Older adults

## Abstract

**Background:**

Poverty, as a risk factor for loneliness, has been understudied, and there is a need to gain a better understanding of the relationship between poverty examined by material deprivation and loneliness among older adults in Hong Kong. It also aimed to explore the possible mediation and moderation effects of social support, social networks, neighborhood collective efficacy, and social engagement in the link between material deprivation and loneliness.

**Methods:**

1696 Chinese older adults aged 60 years and above (*M*_*age*_ = 74.61; *SD* = 8.71) participated in a two-wave study. Older adults reported their loneliness level, material deprivation, perceived level of social support, social network, neighborhood collective efficacy, social engagement, and sociodemographic information. Logistic regression was conducted to examine the effect of material deprivation on loneliness, as well as the mediation and moderation models.

**Results:**

The results indicated that material deprived older adults reported a significantly higher level of loneliness 2 years later when controlling for demographic variables, health-related factors, and loneliness at baseline. We also found that engagement in cultural activities partially mediated the effect of material deprivation and loneliness. Furthermore, neighborhood collective efficacy and engagement in cultural activities were significant moderators that buffer the relationship between material deprivation and loneliness.

**Conclusions:**

Our results suggested the need to alleviate the negative impact of material deprivation on loneliness by developing interventions focused on promoting neighborhood collective efficacy and social engagement, which could be aimed at building meaningful bonds among Chinese older adults in Hong Kong.

**Supplementary Information:**

The online version contains supplementary material available at 10.1186/s12877-024-05013-1.

## Introduction

Loneliness is a pervasive issue among older adults, and it is a significant public health concern that cannot be underestimated. As the population ages, the number of older adults experiencing loneliness is increasing, which has a significant impact on their overall well-being and quality of life. Loneliness has been associated with adverse physical and mental health outcomes, such as increased blood pressure, diabetes, cardiovascular disorder, depressive symptoms, cognitive impairment, Alzheimer’s disease, suicide attempts, and mortality [1, 2]. Previous studies have established that various risk factors like sociodemographic, physical functioning, mental well-being, social relationships, and social engagement contribute to loneliness in older adults [1–3].

Compared with other measures of poverty, material deprivation is a better assessment that represents the living standards older adults are experiencing. Previous studies found that material deprivation is a better predictor of life satisfaction and depression than income-based measures of poverty [4, 5]. In line with other studies, material deprivation measurements, compared to income-based poverty indicators, are a better predictor for health status measured using health outcomes [6].

Previous studies have found varying mediating and moderating roles of social support, social networks, neighborhood collective efficacy, and social engagement in the association of socioeconomic position, wealth, or economic hardship with loneliness [7–9]. No study has been conducted to examine the mediating and moderating role of social support, social networks, neighborhood collective efficacy, and social engagement in the relationship between material deprivation and loneliness. To address these two research gaps, our study aimed to examine the relationship between material deprivation and loneliness in older adults and delineate the mediating and moderating roles of social support, social networks, neighborhood collective efficacy, and social engagement in the association between material deprivation and loneliness.

### Prevalence of loneliness

In 2021, amidst the COVID-19 pandemic, the World Health Organization declared loneliness a significant health concern among older adults [10]. *Loneliness* is a multidimensional and complex construct that comprises social, emotional, and existential loneliness and is defined as a negative feeling due to discrepancies between desired and actual social relationships [11]. Loneliness in older adults is approximately 31% in Europe, 43% in the United States, and 25% in China [12, 13]. A longitudinal study examining social exclusion in Hong Kong found that among 1686 older adults, 46% felt lonely sometimes or most of the time [14].

### Risk factors of loneliness

Loneliness is influenced by risk factors such as sociodemographics, physical functioning, mental well-being, social relationships, and social engagement. Regarding sociodemographics, loneliness is associated with age, gender, marital status, household income, and assets [1, 15, 16]. Previous studies have found that women who are older and not married are more likely to report a greater sense of loneliness compared to men who are in their early aging years, married or widowed [16]. Studies conducted in the United States and China have shown that older adults residing in low or medium-to-high-income households have reported feeling more lonely than those in high-income households [17, 18]. Loneliness is also influenced by an individual’s health, such as depression, self-perceived health, sleep quality, and functional health [1, 19–21].

Recent studies suggested that poor self-rated health, sleep issues such as shorter duration, poor quality sleep, and limitations in functional health are all associated with an increased likelihood of loneliness among older adults [22]. Social relationships and engagement risk factors include social contacts, social networks, social support, social participation, and quality of relationships [1, 17, 21]. Recent studies have indicated that individuals with smaller social networks, fewer sources of support, and lower levels of social engagement in activities are associated with an increased risk of loneliness [8, 17, 23].

### Poverty and loneliness

In Hong Kong, the proportion of older adults aged 65 and above is projected to increase from 21.9% in 2023 to 36% by 2046 [24]. Of which, at least 45% have been living in poverty as of 2020. The increasing proportion of the aging population highlights the importance of tackling poverty-related issues in Hong Kong. Along with inadequate retirement protection, older adults may choose to remain in the labor market until they are forced to retire, face financial hardship, or live in poverty [25]. Previous studies have shown that loneliness is closely linked to socioeconomic factors, with those in disadvantaged financial situations being more prone to loneliness, affecting their social resources [26–28]. The high poverty rate among older adults will continue to increase in tandem with the aging population. Thus, it is crucial to investigate the impact of poverty on loneliness among older adults in Hong Kong.

### Theoretical framework

**Fig. 1:**
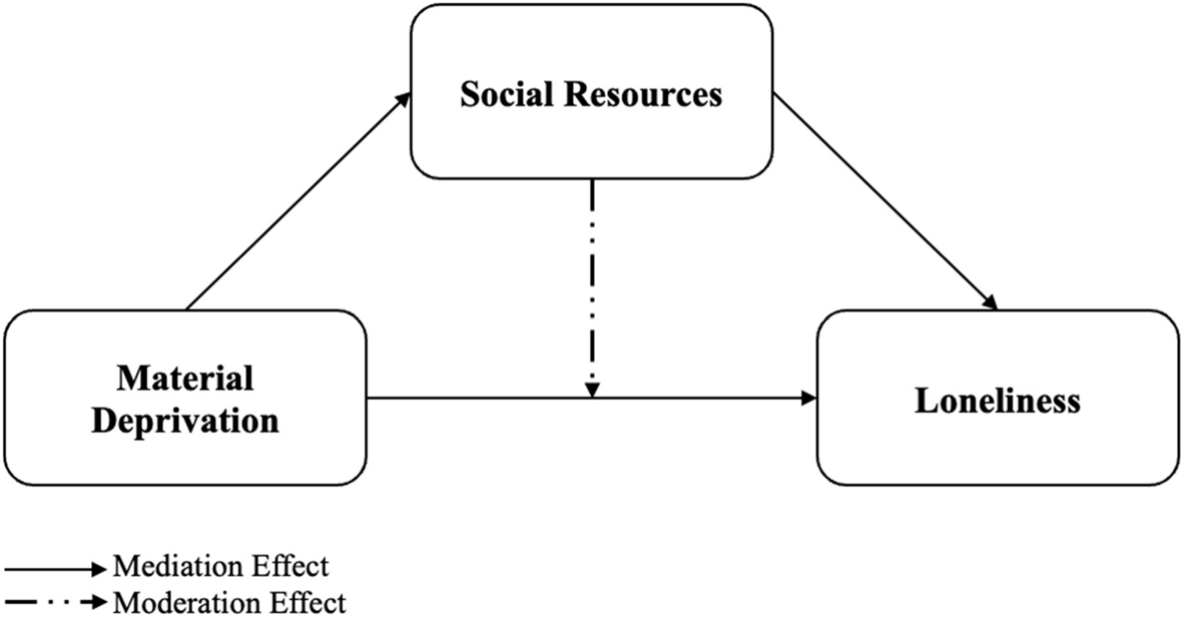
Theoretical framework for current study

The stress process theory, which was first proposed in the 1980s, placed a strong emphasis on the contribution of social structures and experiences in the development of stress, with a focus on how social roles and statuses affect exposure to stressors [29, 30]. It further suggests that life events, chronic strains, and societal structures can lead to differential exposure to stressors and subsequent adverse mental and physical health outcomes [30, 31]. Notably, stressors are not separate events; rather, they are interconnected. For instance, the connection through which disruptive job events led to income loss and economic strain, which in turn result in depression [30]. The stress process theory was further enhanced by a comprehensive conceptual model that highlighted the presence of mediators and moderators that could affect the relationship between the stressor and mental health outcomes [32]. Previous studies using the stress process theory found that older adults with a lower social standing experience more stress, which increases their likelihood of developing health issues, and that social resources can help prevent or lessen the negative impacts of stress on one’s health [33]. Likewise, older adults with lower socioeconomic standing often have fewer resources to deal with pressures. Thus, the current study will build on the stress process theory to examine the direct effect of material deprivation on loneliness and investigate the mediating and moderating role of social resources in the link between material deprivation and loneliness (see Fig. 1).

### Poverty indicators

Several measures are used to assess the poverty situation of older adults. Income-, expenditure-, and asset-based poverty have been used to examine the poverty of Hong Kong older adults [5], while material deprivation is another measure that focuses on assessing whether an older adult’s material well-being is fulfilled through their ability to afford basic needs, such as finances, healthcare, housing, and daily necessities [34, 35]. Material deprivation assesses the essential items that an individual lacks without the need to account for the cost of living and various financial challenges experienced by different people [36]. This approach acknowledges the diverse abilities of older adults to access alternative resources, such as tapping into their personal assets, incurring debt, or seeking financial assistance from others to meet their basic needs [35]. In this study, we focus on material deprivation because it is a good indicator of the living standard of older adults. Recent studies in Hong Kong found that material deprivation has a stronger association with depressive symptoms and life satisfaction than other measures of poverty, such as income- or asset-based measures [4, 5]. Previous studies in other cultures used material deprivation as a proxy for poverty and found a significant association with health outcomes [6, 37, 38]. Hence, this study aims to investigate the association of material deprivation with loneliness among Hong Kong Chinese older adults using a two-year longitudinal data.

### Social resources

Although social resources have often been defined broadly and interchangeably, these terms are, in fact, distinct perspectives in understanding the influence of social relationships on oneself [39]. Social support highlights the importance of both formal and informal assistance that is perceived to be accessible to them; (ii) social network emphasizes the close relationship within individual’s social ties; and (iii) social integration focuses on the individual’s social participation (e.g., engagement in social activities, sense of community) [40, 41]. Social participation can be classified using the taxonomy of social activities as an individual’s degree of engagement by oneself, with others, or for others [42]. Previous studies have established the association between loneliness and social indicators such as income, health, and living conditions [43–45], which have been linked to poor and vulnerable older adults [43]. Social support, social networks, neighborhood collective efficacy, and social participation are all directly associated with loneliness [9, 15, 46]. Research in Portugal noted that social support from family, friends, or significant others mediated the relationship between disadvantaged socioeconomic positions and the quality of life of older adults [7]. Turning to moderating factors, a study in the United States suggested that social support can moderate the effect of involuntary retirement, a socioeconomic disposition, on loneliness among older adults [47]. Besides, social networks were a significant moderator in the link between household indebtedness and depressive symptoms among older adults in China [48]. Social participation cushions the risk of loneliness through gender and wealth, particularly for older adults who participated less frequently in social activities in Europe [9]. Neighborhood collective efficacy was reported to be a significant moderator between material deprivation and depressive symptoms in Hong Kong older adults [49]. Using stress process theory, social resources (i.e., social support, engagement in cultural activities, neighborhood collective efficacy, and social network), which are well-known risk factors for loneliness, may serve both functions either as a mediator or moderator [50]. Regarding mediators, materially deprived older adults may have weakened social resources, leading to a higher level of loneliness. As for moderators, the strength of social resources may reduce the relationship between material deprivation and loneliness. Hence, this study will examine the relationship of it.

### The present study

Based on the theoretical framework and evidence, the present exploratory study tries to answer the following research question: (1) Is material deprivation directly associated with loneliness? (2) What social resources mediate the relationship between material deprivation and loneliness? and (3) What social resources moderate the relation between material deprivation and loneliness?

## Methods

### Sampling and sample size

The current study used two waves of secondary data collected in 2015 and 2017 among older adults aged 60 and older in Hong Kong. A stratified sampling design was used to randomly select older adults based on (i) living quarters and (2) at least one household member aged 60 years and above. Using logistic regression in G*Power 3 [51], with 95% power, the minimum sample size for this study was 739 at a 5% significance level. A total of 2852 households were successfully visited and recruited to join the study in 2015. Participants 60 years and above were the only inclusion for this study. Informed consent was sought after participants were informed and met the requirements of the study. Face-to-face interviews were conducted by well-trained research assistants using a structured questionnaire, and interrater reliability was established through supervision and monitoring. Upon completion of each timepoint, participants were given cash coupons (HKD100 ≈ USD12) for their participation. The second wave of data was collected in 2017, and 1696 of the original participants were successfully contacted and interviewed, resulting in an attrition rate of 40.5%.

### Measures

#### Dependent variable

*Loneliness.* Participants were asked to indicate how often they felt lonely using a 5-point Likert scale (ranging from 0 = never to 4 = always). A single-item question has been widely used in studies of loneliness and good validity has been reported [16, 19, 52, 53]. Previous studies have used a single-item scale to identify the risk factors of loneliness [16, 45, 54]. Following the procedure of previous studies [52, 55, 56], the item was transformed into a dichotomous variable in this study, which reflected not lonely (responses ranging from never to hardly) and lonely (response ranging from sometimes to always). This approach was comparable to the UCLA and De Jong Gierveld loneliness scale, which was strongly associated with mental health [56].

#### Independent variable

*Material Deprivation.* The Material Deprivation Index [57], translated and validated in Hong Kong by Chou and Lee (2018), was utilized in this study. The scale comprised of 28 items and covered five key areas: accommodation, food and clothing, medical care, social connections, and basic amenities. For each item, the participants were asked to indicate whether they (i) possessed it or (ii) did not possess it because they could not afford it. The threshold of material deprivation was established if the participants lacked at least five or more of the essential items due to their inability to afford them.

#### Mediators and/or moderators

*Engagement in Cultural Activities.* The measure of engagement in cultural activities was adopted with minor modifications from the English Longitudinal Study of Ageing [58] to measure social participation among older adults. Participants were asked about the frequency of their participation *in (i)the cinema; (ii)an art gallery or museum; (iii) the theatre, concert, or opera; (iv) restaurants, cafes, or pubs; and (v) Mainland China or abroad?’.* Participants rated their frequency on a 6-point Likert scale (0 = *not at all*, 5 = *twice or more per month*). Given the small sample size in two categories, we collapsed them into a 4-point scale with an overall score of 3. Engagement in cultural activities was calculated based on the average of the five items. The Cronbach’s alpha for these five items was 0.74.

#### Neighborhood collective efficacy

The Neighborhood Collective Efficacy Scale was modified and measured using eight statements (Sampson et al. [59]. The scale has been validated and widely used in the Chinese population [5, 60]. The eight statements focused on social cohesion and informal social control in the individual’s neighborhood. Statements include *‘This is a close-knitted area’, ‘People in the neighborhood are willing to help each other’,* and *‘People in the area can be trusted’*. The responses were rated on a 5-point Likert scale (1 = *strongly agree*; 5 = *strongly disagree*). Statements that were negatively worded were reverse-coded. A mean score was calculated, with a higher score indicating more social control and higher social cohesion and trust. The Cronbach’s alpha for the present study was 0.77.

#### Social support

Participants were asked to gauge the social support they received using three items extracted from The Lubben Social Network Scale [61], which was widely used with the aging population in Hong Kong [62]. The questions included ‘Do you have someone who looks after you when you are sick and have to stay in bed for a few days?’, ‘Do you have someone who can lend you $3000 when you have an urgent need?’ and ‘Do you have someone to give you advice when making an important decision?’. Questions were rated 0 = no and 1 = yes. The social support score was calculated by summing all three items. The Cronbach’s alpha of the three items was 0.85.

#### Social network

Participants were asked to respond to the following three questions on their network: *‘How many of your children do you feel close to?’, ‘How many of your relatives do you feel close to?’* and *‘How many of your friends do you feel close to?’*. Each question was rated continuously and represented three different variables: number of close children, number of close relatives, and number of close friends, respectively. The total social network size was also computed by summing the available network based on the three questions related to their personal network [63].

#### Sociodemographic and covariates

*Sleep Quality.* Participants were asked how refreshed they felt after their sleep [4], on a 4-point Likert scale (1 = *very good*; 4 = *very bad)*.

#### Self-rated health

Participants rated their current health on a scale ranging from 1 = very good to 5 = very poor [64]. The response was recoded to 0 = good health (ranging from very good to average) and 1 = poor (ranging from poor to very poor).

#### Activities of daily living

The Barthel Index of Activities of Daily Living (ADL) asked participants how capable they were at performing 10 different tasks: feeding, transfers, dressing, bowels, bladder, grooming, mobility, toilet use, bathing, and stairs. Participants rated the items from 0 = dependent to 5/10/15 = independent. The total scores were calculated by tabulating all items, with some items being reverse-coded; a higher score indicated a higher level of independence. The Cronbach’s alpha for the scale was 0.59.

#### Instrumental activities of daily living

Participants rated their independent living skills using the Lawton Instrumental Activities of Daily Living (IADL) Scale. The categories included the ability to use the telephone, shop, food preparation, housekeeping, laundry, mode of transportation, responsibility for one’s medications, and handling finances. Participants rated each item either 0 = cannot do it or 1 = able to do it. The sum score ranged from 0 to 8, with a higher score indicating a higher capability to carry out independent living activities. The Cronbach’s alpha for these eight items was 0.80.

#### Demographics

Participants were asked about their demographic status, and these variables included age, gender, education, and marital status.

#### Data analysis

Data analyses were conducted using IBM SPSS Statistics Version 29. Firstly, descriptive was used to describe the general characteristics of our sample. Given the high attrition rate (40.5%), a formal attrition analysis was conducted on all variables between the dropouts and survivors of this study, using t-test for continuous variables and Chi-square test for categorical variables. Logistics regression was used to examine the direct effect of material deprivation and loneliness. The guidelines suggested by Baron and Kenny [65] were employed to assess the mediating and moderating effects in the study.

## Results

### Characteristics of respondents

Table 1 shows the descriptive statistics of the participants. At baseline, data was collected among 2852 participants (*M*_*age*_ = 74.31, *SD* = 8.76). Most of the older adults were female (54.1%), married (54.5%), and had primary school education (45.8%). This study sample comprised 1696 participants (*M*_*age*_ = 74.61, *SD* = 8.71). The majority were female (53.9%), married (53.2%), and had primary education (45.4%). An attrition analysis was performed, and the sample quality between the dropouts and survivors was unaffected. Sleep quality (*M*_*T2*_ = 1.70 vs *M*_*T1*_ = 1.68, *p* < 0.05) was poorer among survivors than dropouts. Among our study sample, one-third reported being materially deprived and a higher level of loneliness at T1 (71.4%) compared to T2 (64.0%).

**Table 1:** Characteristics of Sample (N = 1696)

	Descriptive		Attrition analysis			
	Baseline (N_T1_ = 2852)	Study Sample (*N* = 1696)	Baseline (%, mean)			
	Percentage/ Mean (SD)		Dropouts (*n* = 1156)	Survivors (*n* = 1696)	Chi-square/ t-test	*P*-Value
Loneliness at T2 (%)	–	64.0	–	–	–	–
Material Deprived (%)	29.4	30.0	28.5	30.0	0.80	0.37
Loneliness at Baseline (%)	70.2	71.4	68.5	71.4	2.67	0.10
Age: 60–69 (%)	34.1	32.8	36.0	32.8	5.00	0.08
Gender: Male (%)	45.9	46.1	45.5	46.1	0.10	0.75
Marital Status: Married (%)	54.5	53.2	52.0	53.2	0.69	0.71
Education: Below Primary (%)	34.3	34.5	33.9	34.5	0.35	0.84
Sleep Quality	1.68 (0.66)	1.70 (0.65)	1.64*	1.70*	−2.31	0.02
Poor Self-Rated Health (%)	22.3	22.5	22	22.5	0.11	0.75
Activities of Daily Living	2.79 (7.00)	2.78 (6.59)	2.81	2.78	0.09	0.93
Instrumental Activities of Daily Living	13.21 (2.23)	7.73 (0.92)	7.73	7.73	0.04	0.97
Engagement in Cultural Activities	1.38 (0.64)	1.37 (0.65)	1.41	1.37	1.71	0.63
Neighbourhood Collective Efficacy	3.23 (0.44)	3.22 (0.46)	3.25	3.22	1.42	0.16
Social Support	2.47 (1.01)	2.45 (1.02)	2.51	2.45	1.67	0.10
Number of Close Children	1.99 (1.59)	2.01 (1.60)	1.97	2.01	−0.52	0.61
Number of Close Relatives	4.05 (3.70)	4.02 (3.70)	4.09	4.02	0.53	0.60
Number of Close Friends	2.16 (1.67)	2.12 (1.69)	2.22	2.12	1.60	0.11
Sum of Social Network	8.21 (5.40)	8.15 (5.42)	8.29	8.15	0.71	0.99

Attrition analysis: categorical variables (chi-square test), continuous variables (t-test)

### Correlations of variables with loneliness

The bivariate correlations between study variables and loneliness at T2 are shown in Table 2. The correlation analyses shown that loneliness at T2 was positively associated with material deprivation (r = 0.12, *p* < 0.001), age (r = 0.17, p < 0.001), marital status (r = 0.16, *p* < 0.001), sleep quality (r = 0.10, p < 0.001), self-rated health (r = 0.15, p < 0.001), ADL (r = 0.14, p < 0.001), engagement in cultural activities (r = 0.11, p < 0.001) and negatively associated with education (r = − 0.13, *p* < 0.001), neighborhood collective efficacy (r = − 0.27, p < 0.001), social support (r = − 0.08, *p* = 0.001), and number of close friends (r = − 0.10, p < 0.001). Loneliness at T2 was highly associated with loneliness at baseline (r = 0.62, p < 0.001); thus, it was necessary to control it in the analysis. The strength between the independent variables used in the analysis was moderate in some pairs (| *r* | ≤ 0.45). The number of relatives positively correlated to the number of close children (*r* = 0.64, *p* < 0.001).

**Table 2:**
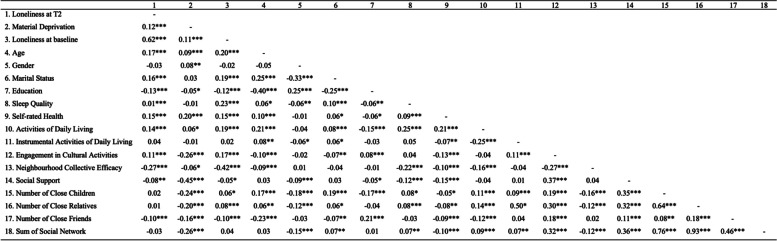
Correlation of all variables with loneliness (*N* = 1696)

*p<0.05, **p<0.01, ***p<0.001

### Association between material deprivation and loneliness

Loneliness was significantly associated with material deprivation, as measured by odd ratios, 1.77 (*95% CI* = 1.41, 2.23). Although significant, the strength of the association between material deprivation and loneliness decreased when we adjusted for loneliness at T1 (*OR* = 1.52, *95% CI* = 1.14, 2.03) and sociodemographic, sleep, self-rated health, ADL, and loneliness at T1 (*OR* = 1.37, *95% CI* = 1.02, 1.85). As seen in Table 3, marital status (*OR* = 1.29, *95% CI* = 1.01, 1.65), sleep (*OR* = 0.78, *95% CI* = 0.63, 0.97), self-rated health (*OR* = 1.47, *95% CI* = 1.05, 2.07), and loneliness at T1 (*OR* = 22.08, *95% CI* = 16.26, 29.99) were significantly associated with loneliness at T2. While gender was not significantly correlated to loneliness at T2, we incorporated it into our analysis as an essential demographic control. Notably, the results were comparable with or without gender being included as a covariate.

**Table 3:** Result of logistic regression on loneliness showing main and mediation effects (*N* = 1696)

	Main Effects			Mediation Effect
	Model 1	Model 2	Model 3	Model 4
	*Odd Ratio*	*Odd Ratio*	*Odd Ratio*	*Odd Ratio*
Material Deprivation	1.77***	1.52**	1.37*	1.31*
*Covariates*				
Loneliness at Baseline		23.24***	22.08***	20.33***
Age			1.07	1.09
Gender			1.02	1.03
Marital Status			1.29*	1.33*
Education			0.85	0.83
Sleep			0.78*	0.77*
Self-Rated Health			1.47*	1.56*
Activities of Daily Living			1.01	1.01
*Mediator*				
Engagement				1.24*
Model Coefficients	χ2(1) = 25.17***	χ2(2) = 669.19***	χ2(9) = 667.17***	χ2(10) = 672.44***
Nagelkerke R^2^	0.02	0.45	0.46	0.46

*p < 0.05, ** *p* < 0.01, *** *p* < 0.001

Model 1 Adjusted for material deprivation

Model 2 Adjusted for material deprivation and loneliness at baseline

Model 3 Adjusted for material deprivation, loneliness at baseline, age, gender, marital status, education, sleep, self-rated health, and activities of daily living

### Mediation model

Mediating variables (i.e., social support, engagement in cultural activities, neighborhood collective efficacy, and social network) included in the analysis were correlated with loneliness at T2 and material deprivation (see Table 2). Using Baron & Kenny’s [65] guidelines, the mediation effect must meet the following conditions: (i) a significant association between material deprivation and loneliness, (ii) a significant association between material deprivation and mediator, (iii) a significant association between mediator and loneliness, and (iv) when controlling for material deprivation, a significant association was found between the mediator variable and loneliness, and the association between material deprivation and loneliness becomes (a) non-significant indicates the presence of a mediation effect, or (b) reducing significance suggests the presence of a partial mediation effect.

For social support, the results fulfilled condition 1, where the association between material deprivation and loneliness was significant (OR = 1.77, *p* < 0.001); condition 2 where there was a significant association between material deprivation and social support (B = − 0.45, p < 0.001), and condition 3 where social support and loneliness was significantly associated (OR = 0.84, p < 0.001). However, in condition 4, a non-significant association was found between social support and loneliness (OR = 0.89, *p* > 0.05) and material deprivation and loneliness (OR = 1.23, p > 0.05) (see Supplementary Table S1). Hence, social support was not a mediator. The model was repeated for engagement in cultural activities, neighborhood collective efficacy, and the number of close friends. However, neighborhood collective efficacy and social network (i.e., number of close friends) were found to be non-significant mediators (see Supplementary Table S1). In terms of engagement in cultural activities, the results indicated that (i) material deprivation and loneliness were significant (OR = 1.77, *p* < 0.001), (ii) significant association between material deprivation and engagement in cultural activities (B = − 0.28, p < 0.001), (iii) significant association between engagement in cultural activities and loneliness (OR = 1.36, p < 0.001). In the final condition, the effects between material deprivation and loneliness (OR = 1.31, *p* < 0.05) were significant after controlling for engagement in cultural activities (OR = 1.24, p < 0.05), indicating that engagement in cultural activities was a partial mediator.

### Moderation model

**Fig. 2:**
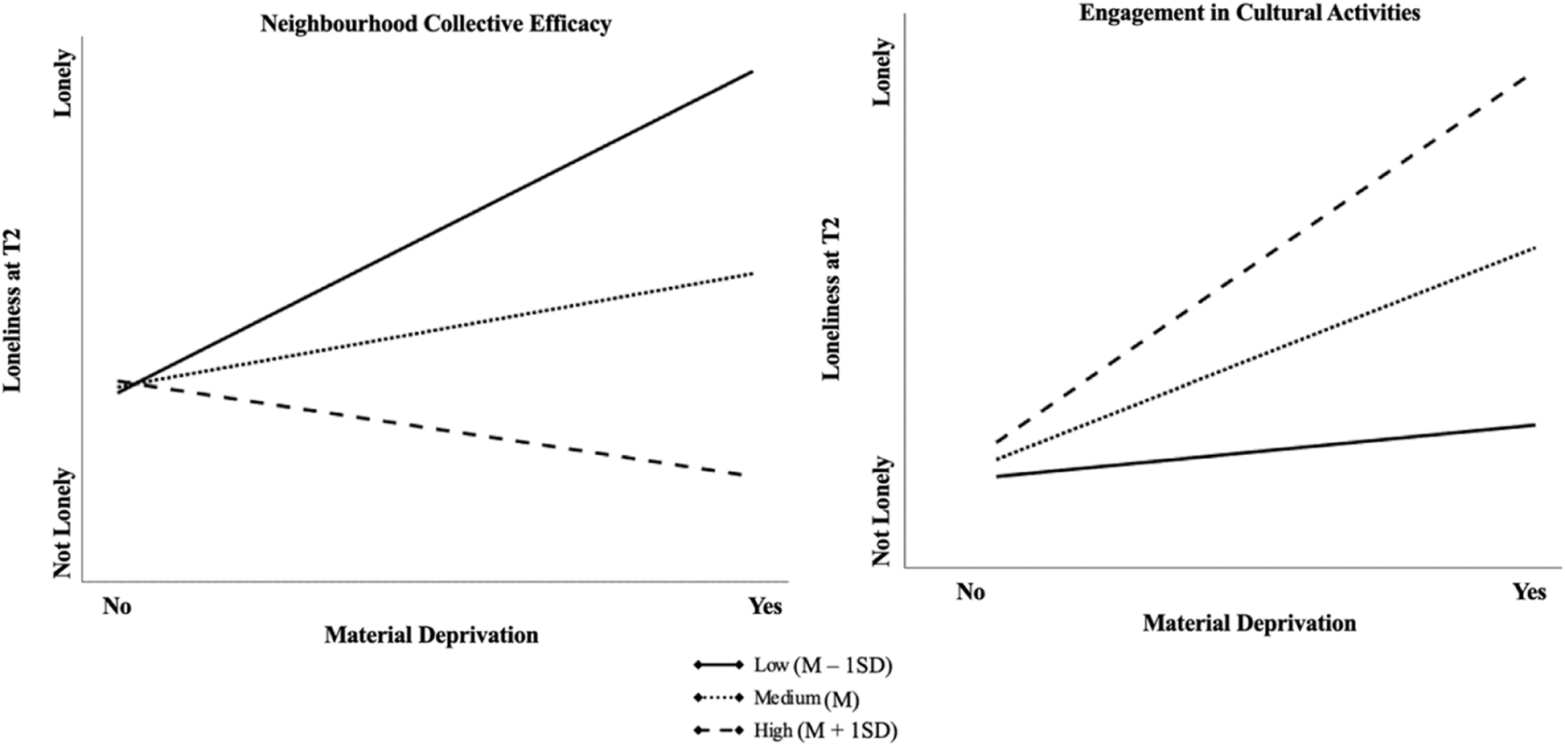
Moderation effect of engagement in cultural activities and neighborhood collective efficacy (*N* = 1696)

Moderation effects were examined using Baron & Kenny’s [65] guidelines, where an interaction term was added between material deprivation and the potential moderator into the baseline model. Two significant interaction terms between engagement in cultural activities and material deprivation (*OR* = 1.56, *p* < 0.05) and neighborhood collective efficacy and material deprivation (*OR* = 0.33, *p* < 0.01) on loneliness (see Table 4). Figure 2 represents the interaction effect of engagement in cultural activities and neighborhood collective efficacy between material deprivation and loneliness. The findings suggested that older adults who engaged in medium to high levels of engagement in cultural activities helped to buffer the effect of material deprivation on loneliness, especially those who are material deprived. Furthermore, material deprived older adults with low neighbourhood collective efficacy exhibited higher levels of loneliness. However, the terms of interaction for social support and the number of close friends were not found to have any moderating effect on material deprivation and loneliness (see Supplementary Table S1).

**Table 4:** Result of logistics regression on loneliness showing moderation effects (*N* = 1696)

	Moderation Effects	
	Model 5	Model 6
	*Odd Ratio*	*Odd Ratio*
Material Deprivation	0.82	48.78**
*Covariates*		
Loneliness at Baseline	19.95***	21.66***
Age	1.08	1.08
Gender	1.02	1.00
Marital Status	1.33*	1.28*
Education	0.84	0.86
Sleep	0.78*	0.74**
Self-Rated Health	1.57*	1.48*
Activities of Daily Living	1.01	1.00
*Moderator*		
Engagement	1.11	
Neighborhood		1.03
*Interaction Term*		
MD X Engagement	1.56*	
MD X Neighborhood		0.33**
Model Coefficients	χ2(11) = 677.06***	χ2(11) = 666.84***
Nagelkerke R^2^	0.46	0.46

*p < 0.05, ** *p* < 0.01, *** *p* < 0.001

*Engagement* Engagement in Cultural Activities, *Neighborhood* Neighborhood Collective Efficacy, *MD* Material Deprivation

Model 5 Adjusted for material deprivation, loneliness at baseline, age, gender, marital status, education, sleep, self-rated health, and activities of daily living, engagement, and interaction term (MD X Engagement)

Model 6 Adjusted for material deprivation, loneliness at baseline, age, gender, marital status, education, sleep, self-rated health, and activities of daily living, neighborhood, and interaction term (MD X Neighborhood)

## Discussion

Understanding the impact of material deprivation on loneliness for older adults is crucial in developing and implementing prevention, intervention, and treatment measures to combat loneliness in old age and alleviate the impact of living in poverty on loneliness. It is critical to monitor the loneliness and poverty situation, supplement existing poverty measures, and develop initiatives to curb loneliness among older adults in Hong Kong. Given that poverty is understudied with loneliness, this study is one of the first to (1) explore the plausible association between material deprivation and loneliness in Chinese older adults and (2) assess mediating and moderating social resources, such as social support, social network, neighborhood collective efficacy, and engagement in cultural activities, in the link between material deprivation and loneliness. Our findings revealed that material deprivation directly impacts loneliness among older adults. Engagement in cultural activities partially mediated the effect of material deprivation on loneliness, and neighborhood collective efficacy and engagement in cultural activities moderated the impact of material deprivation and loneliness.

Within the context of Hong Kong, this is the first time material deprivation has been explored with loneliness. The rate of material deprivation is comparable to the poverty rate reported. Our findings indicate that older adults who are materially deprived are more likely to report feeling lonely 2 years later after controlling for demographic variables, sleep quality, self-rated health, ADL, and loneliness at baseline. This finding is in line with the stress process theory, suggesting that material deprivation is a stressor that leads to mental health issues, which in our study is loneliness [30]. A recent study in the Netherlands found that subjective debt burden is a significant predictor of loneliness in older adults, suggesting that older adults are less likely to meet their living standard due to their economic situations [66]. As the poverty rate continues to rise in Hong Kong, older adults are placed in a vulnerable position where they not only have to worry about their daily necessities but also about psychological distress, which leads to loneliness and other mental health outcomes. Scholars highlighted that material deprivation is also associated with more significant depressive symptoms and lower life satisfaction [5]. The official poverty indicator in Hong Kong is defined as half of the median household income adjusted by the household size; this highlights the limitation that the Hong Kong SAR Government only uses income to differentiate between individuals in poverty and those not [25]. Our findings suggest that, on top of the existing income-based measure, policymakers should consider complementing it with measures of material deprivation to reflect the actual needs and resources of the older population [67].

Material deprivation plays a substantial role as a risk factor in contributing to loneliness among older adults. This implies that material deprivation extends beyond mere financial difficulties and includes access to adequate resources for daily living. Being materially deprived can cause older adults to feel financially stressed as they must prioritize their basic needs over social interactions and restrict their social participation due to financial constraints or the stigmatism that arises from a lack of social acceptance. Hence, future research should continue to explore material deprivation as a potential risk factor in (i) other aspects of mental health, such as anxiety, depression, or suicidal ideation; (ii) physical health in terms of self-rated health, chronic illnesses, and functional capacity; and (iii) quality of life. Other poverty indicators, such as income-, expenditure-, and asset-based measures, with loneliness among older adults could also be explored.

In addition to the significant impact of material deprivation on loneliness, other factors, such as loneliness at baseline, marital status, sleep quality, and self-rated health, were found to have a significant impact on loneliness. These findings are consistent with previous findings [1, 16, 19, 20]. Specifically, self-rated health (*OR* = 1.47) had a higher odd ratio than material deprivation (*OR* = 1.37). Previous studies have highlighted the association between self-rated health and loneliness in older adults [54]. Furthermore, a stronger association between self-rated health and socioeconomic status was observed in lonely older adults [3, 28]. Given that self-rated health is a subjective measurement, older adults experiencing loneliness might report a poorer health status even if they are not experiencing any health symptoms.

Another contribution of this study is that engagement in cultural activities partially mediated the effect of material deprivation on loneliness in older adults. According to the social stress theory, social resources may help to shape the relationship between stress and mental outcomes. Older adults who participate in social activities are less likely to feel lonely due to the individual’s social participation taxonomy based on their level of involvement and the goal of the activities [17, 23, 42]. The finding further suggests that as engagement in cultural activities increases, the impact of material deprivation on loneliness decreases. A plausible explanation is that materially deprived older adults carefully choose the type of social activities they can participate in to reduce the impact of material deprivation on loneliness. These activities could be low-cost but increase social connections and social support through participation [44]. Thus, it is vital to promote engagement in cultural activities among older adults, and social service providers could develop activities to facilitate participation in cultural activities.

Engagement in cultural activities significantly interacts with material deprivation and loneliness, suggesting that participating in social activities may weaken the effect of loneliness, especially for materially deprived older adults. We expected that social engagement would buffer the effect of loneliness, where materially deprived older adults who experience a higher level of loneliness participate less in social activities [9, 26]. In our study, material deprivation is associated with increased loneliness, and medium to high engagement in cultural activities significantly enhanced the effect of loneliness. A possible explanation for this finding is that materially deprived older adults might be participating in cultural activities that are single-person oriented, which does not promote interaction between others, or may not be able to make reliable friendships in group activities, increasing loneliness. Further investigation is needed. For now, community-organized events should aim at creating an interactive platform where older adults can build meaningful bonds with each other.

Neighborhood collective efficacy can buffer the impact of loneliness among older adults who are materially deprived. The stress process theory suggests the importance of focusing on the availability of resources in the larger community to help lessen the effect of chronic strain on health outcomes. Higher neighborhood collective efficacy is often associated with strong social bonds and support networks, which can help older adults experiencing material deprivation feel more connected and less lonely, even in the face of financial hardship. Our findings are consistent with the previous findings, where neighborhood collective efficacy was found to be more beneficial for the well-being of older adults living in deprived conditions [9]. This suggests that older adults who are materially deprived receive better support from the community. Thus, for older adults with low neighborhood collective efficacy, interventions focusing on reducing material deprivation might significantly impact reducing loneliness, and for those with high neighborhood collective efficacy, enhancing neighborhood support could be an effective way to combat loneliness in the face of material deprivation.

Our study contradicts previous findings where social support and social networks were neither mediators nor moderators between socioeconomic status and mental health outcomes. Previous studies found mediating effects utilized socioeconomic well-being or wealth as a proxy measurement of poverty, while our study specifically focused on material deprivation. The constructs within socioeconomic, wealth, and poverty measurements might differ substantially, resulting in different results. A plausible reason why social support did not mediate, or moderate could be related to the measurement not being comprehensive, whereby our study focused on the perceived social support instead of the receipt of emotional support. Furthermore, the number of social networks does not necessarily translate into the quality of relationships, as adult children matter more to older Chinese adults than their friends and relatives [68]. Older adults might be uncomfortable disclosing sensitive information regarding their hardships to others outside their immediate network. Furthermore, only the size of the network was included in this study, not the frequency and quality of contacts within the network. Hence, future studies could explore the size, frequency, and quality of social networks among older adults.

### Limitations and future direction

The current study has several limitations that warrant attention when interpreting our results and considering future research. The fact that only two waves of data were included in the longitudinal study limits the ability to conclude the long-term relationship between material deprivation and loneliness. Future studies could include more than two waves to ensure consistency in the results. Changes in poverty status and loneliness within 2 years are possible; more frequent assessment may provide more insights into identifying risk factors. Rather than using a standardized scale, this study used a single-item scale to measure loneliness, which may not capture the complexity of the construct and raises questions regarding its reliability. However, many studies that used a single-item scale to identify the risk factors of loneliness in older adults reported good validity and reliability [16, 45, 54]. Consideration of cultural and social differences is necessary as a single-item scale could allow older adults to understand the concept of loneliness better. Given the scarcity of longitudinal data, this study is worthwhile as it provides exploratory insights into poverty and loneliness using secondary data. A high prevalence of loneliness was also observed in our study, which can be attributed to the recoding of the single-item loneliness scale, which was scored on a 5-point Likert scale, to a dichotomous variable, where most older adults were more inclined to select ‘sometimes,’ resulting in them being classified as lonely in our study. As such, future studies could use established scales such as UCLA and the De Jong Gierveld loneliness scale. Another limitation of this study is the high attrition rate. Although our attrition analysis revealed that it did not affect our study, we suggest that future studies could check in with participants between waves to ensure their ongoing participation, provide additional incentives for full study completion, or perform analyses with adjusted weightage [69]. This study only utilized one measure of poverty; other indicators, such as income-based poverty, expenditure-based poverty, asset-based poverty, and social exclusion, could be considered in future research. It is helpful to compare the predictive power of these poverty indicators with material deprivation. The material deprivation index included a subscale of social connection, and this concept may overlap with social resources. However, without the subscale, we cannot delineate the thresholds for material deprivation. Hence, this could be one of the reasons why material deprivation is associated with loneliness, and future studies may further examine this issue by including those social connections as potential mediators in the link between material deprivation and loneliness. Socioeconomic status affects the availability and accessibility of social resources [27]; future studies could examine the impact of different poverty measures with the changes in social resources at individual, family, and community levels. At the individual level, older adults living in poverty will have limited access to social resources, which can negatively affect their overall well-being; at the family level, poverty could lead to increased stress and strain on relationships; and at the community level, poverty could lead to limited participation and increased risk of negative outcomes. Although LSNS 3-item has been widely used, future research might consider using the LSNS-6 to provide a comprehensive insight on social support rendered by family and friends [62]. Despite these limitations, this study provides insights for policymakers, community service providers, and future research to develop interventions and programs to reduce loneliness, mainly to alleviate poverty’s impact on loneliness among older adults.

## Conclusions

This study sheds light on the association between material deprivation and loneliness in older Chinese adults in Hong Kong, an understudied area. Our findings reveal that material deprivation contributes to increased loneliness among older adults and the mediating and moderating role of engagement in cultural activities and neighborhood collective efficacy. These findings underscore the importance of promoting social interaction between older adults and building supportive community environments to alleviate the negative impact of material deprivation on loneliness.

### Supplementary Information

**Supplementary Material 1.**

## Data Availability

Data will be made available on reasonable request from the corresponding author, Vivien F.Y. Tang s1135125@s.eduhk.hk.

## References

[1] Dahlberg L, McKee KJ, Frank A, Naseer M (2022). A systematic review of longitudinal risk factors for loneliness in older adults. Aging Ment Health..

[2] Leigh-Hunt N, Bagguley D, Bash K, Turner V, Turnbull S, Valtorta N, Caan W (2017). An overview of systematic reviews on the public health consequences of social isolation and loneliness. Public Health..

[3] Zuo S, Lin L, Chen S, Wang Z, Tian L, Li H, Xu Y (2023). Influencing factors of loneliness among older adults in China: a systematic review and meta-analysis. Psychogeriat..

[4] Zhu AYF, Chou KL. The effects of multidimensional poverty on life satisfaction among older adults in Hong Kong. J Appl Gerontol. 2023;42(5):1022–34. 10.1177/07334648221141410 36440625

[5] Cheung KCK, Chou KL (2019). Poverty, deprivation and life satisfaction among Hong Kong older persons. Ageing Soc..

[6] Myck M, Najsztub M, Oczkowska M (2020). Implications of social and material deprivation for changes in health of older people. J Aging Health..

[7] Henriques A, Silva S, Severo M, Fraga S, Barros H (2020). Socioeconomic position and quality of life among older people: the mediating role of social support. Prev Med..

[8] Zhao L, Wu L (2022). The association between social participation and loneliness of the Chinese older adults over time—the mediating effect of social support. Int J Environ Res Public Health..

[9] Niedzwiedz CL, Richardson EA, Tunstall H, Shortt NK, Mitchell RJ, Pearce JR (2016). The relationship between wealth and loneliness among older people across Europe: is social participation protective?. Prev Med..

[10] World Health Organization. Social isolation and loneliness among older people: advocacy brief. 2021.

[11] Perlman D, Peplau LA, Duck M, Gilmour R (1981). Towards a social psychology of loneliness. Personal relationships in disorder.

[12] Yang F, Gu D (2021). Widowhood, widowhood duration, and loneliness among older adults in China. Soc Sci Med..

[13] National Academies of sciences E, medicine: social isolation and loneliness in older adults: opportunities for the health care system. Washington, DC: The National Academies Press; 2020. 32510896

[14] Chou KL (2018). Social exclusion in old age: a validation study in Hong Kong. Aging Ment Health..

[15] Cohen-Mansfield J, Hazan H, Lerman Y, Shalom V (2016). Correlates and predictors of loneliness in older-adults: a review of quantitative results informed by qualitative insights. Int Psychogeriatr..

[16] Nyqvist F, Näsman M, Hemberg J, Nygård M. Risk factors for loneliness among older people in a Nordic regional context – a longitudinal study. Ageing Soc. 2021:1–22.

[17] Donovan NJ, Wu Q, Rentz DM, Sperling RA, Marshall GA, Glymour MM (2016). Loneliness, depression and cognitive function in older U.S. adults. Int J Geriatr Psychiatry..

[18] Zhang J, Lu N (2022). How does neighbourhood environment influence loneliness in later life in urban China? The role of financial status. Health Soc Care Commun..

[19] Nicolaisen M, Thorsen K (2014). Who are lonely? Loneliness in different age groups (18-81 years old), using two measures of loneliness. Int J Aging Hum Dev..

[20] Hawkley LC, Cacioppo JT (2010). Loneliness matters: a theoretical and empirical review of consequences and mechanisms. Ann Behav Med..

[21] Hawkley LC, Kocherginsky M (2018). Transitions in loneliness among older adults: a 5-year follow-up in the National Social Life, health, and aging project. Res Aging..

[22] Shankar A (2020). Loneliness and sleep in older adults. Soc Psychiatry Psychiatr Epidemiol..

[23] Holmén K, Furukawa H (2022). Loneliness, health and social network among elderly people - a follow-up study. Arch Gerontol Geriatr..

[24] Census and statistics department: population by sex and age group. In. Edited by Department CaS. Hong Kong; 2023.

[25] Lee S-Y, Chou K-L (2015). Trends in elderly poverty in Hong Kong: a decomposition analysis. Soc Indic Res..

[26] Bosma H, Jansen M, Schefman S, Hajema KJ, Feron F (2015). Lonely at the bottom: a cross-sectional study on being ill, poor, and lonely. Public Health..

[27] Bai Z, Wang Z, Shao T, Qin X, Hu Z (2021). Association between social capital and loneliness among older adults: a cross-sectional study in Anhui Province, China. BMC Geriatr..

[28] Domenech-Abella J, Mundo J, Lara E, Moneta MV, Haro JM, Olaya B (2017). The role of socio-economic status and neighborhood social capital on loneliness among older adults: evidence from the Sant Boi aging study. Soc Psychiatry Psychiatr Epidemiol..

[29] Pearlin LI (1989). The sociological study of stress. J Health Soc Behav..

[30] Pearlin LI, Menaghan EG, Lieberman MA, Mullan JT (1981). The stress process. J Health Soc Behav..

[31] Achdut N, Sarid O (2020). Socio-economic status, self-rated health and mental health: the mediation effect of social participation on early-late midlife and older adults. Isr J Health Policy Res..

[32] Aneshensel CS, Mitchell UA, Johnson RJ, Turner RJ, Link BG (2014). The stress process: its origins, evolution, and future. Sociology of mental health: selected topics from forty years 1970s–2010s.

[33] George LK. Social factors, depression, and aging. Handbook of aging and the social sciences. 2011. p. 149–62.

[34] Verbunt P, Guio A-C (2019). Explaining differences within and between countries in the risk of income poverty and severe material deprivation: comparing single and multilevel analyses. Soc Indic Res..

[35] Nolan B, Whelan CT (2010). Using non-monetary deprivation indicators to analyze poverty and social exclusion: lessons from Europe?. J Policy Anal Manag..

[36] Chou KL, Lee SY (2018). Superimpose material deprivation study on poverty old age people in Hong Kong study. Soc Indic Res..

[37] Doebler S, Glasgow N (2017). Relationships between deprivation and the self-reported health of older people in Northern Ireland. J Aging Health..

[38] Groffen DA, Bosma H, van den Akker M, Kempen GI, van Eijk JT (2008). Material deprivation and health-related dysfunction in older Dutch people: findings from the SMILE study. Eur J Pub Health..

[39] Holt-Lunstad J, Lefler M, Gu D, Dupre ME (2021). Social Integration. Encyclopedia of gerontology and population aging.

[40] Gottlieb BH, Bergen AE (2010). Social support concepts and measures. J Psychosom Res..

[41] Holt-Lunstad J, Uchino B: Social support and health In: Glanz K, Rimer BK, Viswanath K, eds. Health behavior: theory, research, and practice. New York, NY. In.: John Wiley & Sons, Inc; 2015.

[42] Levasseur M, Richard L, Gauvin L, Raymond E (2010). Inventory and analysis of definitions of social participation found in the aging literature: proposed taxonomy of social activities. Soc Sci Med..

[43] Cheung G, Wright-St Clair V, Chacko E, Barak Y (2019). Financial difficulty and biopsychosocial predictors of loneliness: a cross-sectional study of community dwelling older adults. Arch Gerontol Geriatr..

[44] Coll-Planas L, Del Valle GG, Bonilla P, Masat T, Puig T, Monteserin R (2017). Promoting social capital to alleviate loneliness and improve health among older people in Spain. Health Soc Care Commun..

[45] Dahlberg L, Andersson L, Lennartsson C (2018). Long-term predictors of loneliness in old age: results of a 20-year national study. Aging Ment Health..

[46] Kang HW, Park M, Wallace Hernandez JP (2018). The impact of perceived social support, loneliness, and physical activity on quality of life in south Korean older adults. J Sport Health Sci..

[47] Shin O, Park S, Amano T, Kwon E, Kim B (2020). Nature of retirement and loneliness: the moderating roles of social support. J Appl Gerontol..

[48] Gao Q, Prina AM, Prince M, Acosta D, Luisa Sosa A, Guerra M, Huang Y, Jimenez-Velazquez IZ, Llibre Rodriguez JJ, Salas A (2021). Loneliness among older adults in Latin America, China, and India: prevalence, correlates and association with mortality. Int J Public Health..

[49] Cheung KCK, Chou KL (2017). Poverty, deprivation, and depressive symptoms among older adults in Hong Kong. Aging Ment Health..

[50] Pearlin LI, Bierman A. Current issues and future directions in research into the stress process. Handbook of the sociology of mental health. 2013. p. 325–40.

[51] Faul F, Erdfelder E, Lang A-G, Buchner A (2007). G*power 3: a flexible statistical power analysis program for the social, behavioral, and biomedical sciences. Behav Res Methods..

[52] Pinquart M, Sorensen S (2001). Influences on loneliness in older adults: a Meta-analysis. Basic Appl Soc Psychol..

[53] Kotwal AA, Batio S, Wolf MS, Covinsky KE, Yoshino Benavente J, Perissinotto CM, O'Conor RM (2022). Persistent loneliness due to COVID-19 over 18 months of the pandemic: a prospective cohort study. J Am Geriatr Soc..

[54] Zhong BL, Liu XJ, Chen WC, Chiu HF, Conwell Y (2018). Loneliness in Chinese older adults in primary care: prevalence and correlates. Psychogeriat..

[55] Dahlberg L, Agahi N, Lennartsson C (2018). Lonelier than ever? Loneliness of older people over two decades. Arch Gerontol Geriatr..

[56] Kotwal AA, Cenzer IS, Waite LJ, Smith AK, Perissinotto CM, Hawkley LC (2022). A single question assessment of loneliness in older adults during the COVID-19 pandemic: a nationally-representative study. J Am Geriatr Soc..

[57] Townsend P (1979). Poverty in the United Kingdom: a survey of household resources and standards of living.

[58] Barnes M, Blom AG, Cox J, Lessof C, Walker A: The social exclusion of older people: evidence from the first wave of the English longitudinal study of ageing (ELSA), Final report In; 2006.

[59] Sampson RJ, Raudenbush SW, Earls F (1997). Neighborhoods and violent crime: a multilevel study of collective efficacy. Science..

[60] Wang SC, Fowler PJ (2019). Social cohesion, neighborhood collective efficacy, and adolescent subjective well-being in urban and rural Taiwan. Am J Community Psychol..

[61] Lubben JE (1988). Assessing social networks among elderly populations. Family Commun Health..

[62] Chan SCY, Wong CC, Huang QL, Fung CK (2023). The psychometric properties of the Lubben social network scale (LSNS-6) and its associations with well-being indicators in Hong Kong older adults. Australas J Ageing..

[63] Fiori KL, Smith J, Antonucci TC (2007). Social network types among older adults: a multidimensional approach. J Gerontol: Series B..

[64] Liu J, Gou RY, Jones RN, Schmitt EM, Metzger E, Tabloski PA, Arias F, Hshieh TT, Travison TG, Marcantonio ER (2023). Association of loneliness with change in physical and emotional health of older adults during the COVID-19 shutdown. Am J Geriatr Psychiatry..

[65] Baron RM, Kenny DA (1986). The moderator-mediator variable distinction in social psychological research: conceptual, strategic, and statistical considerations. J Pers Soc Psychol..

[66] Loibl C, Drost MA, Huisman M, Suanet B (2022). Bruine de Bruin W, McNair S, summers B: worry about debt is related to social loneliness in older adults in the Netherlands. Ageing Soc..

[67] Aprea M, Raitano M, Silber J (2023). The income and consumption approach to unidimensional poverty measurement. Research handbook on measuring poverty and deprivation.

[68] Thomas PA, Liu H, Umberson D (2017). Family relationships and well-being. Innov Aging..

[69] Teague S, Youssef GJ, Macdonald JA, Sciberras E, Shatte A, Fuller-Tyszkiewicz M, Greenwood C, McIntosh J, Olsson CA, Hutchinson D (2018). Retention strategies in longitudinal cohort studies: a systematic review and meta-analysis. BMC Med Res Methodol..

