# Supplementary material for: An exploratory study on material deprivation and loneliness among older adults in Hong Kong
Source: BMC Geriatr. 2024 May 6;24:400. doi: 10.1186/s12877-024-05013-1 (PMC11071256; doi:10.1186/s12877-024-05013-1)
Supplement: Supplementary file 1 — Supplementary Material 1. [file 12877_2024_5013_MOESM1_ESM.docx]

| **Supplementary Table S1:** Result of logistic regression on loneliness for non-significant mediators & moderators (N=1,696) | | | | | | | | | |
| --- | --- | --- | --- | --- | --- | --- | --- | --- | --- |
|  | **Mediator:**  **Social Support** | **Mediator:**  **Neighborhood** | | **Mediator:**  **Friends** | | **Moderator:**  **Social Support** | | | **Moderator:**  **Friends** |
|  | *Odd Ratio* | | *Odd Ratio* | | *Odd Ratio* | | *Odd Ratio* | *Odd Ratio* | |
| Material Deprived | 1.23 | | 1.33 | | 1.35 | | 0.824 | **1.78*** | |
|  |  | |  | |  | |  |  | |
| *Covariates* |  | |  | |  | |  |  | |
| Loneliness at Baseline | **22.97***** | | **22.79***** | | **22.06***** | | **23.23***** | **22.19***** | |
| Age | 1.06 | | 1.08 | | 1.06 | | 1.05 | 1.06 | |
| Gender | 1.03 | | 1.04 | | 1.01 | | 1.03 | 1.01 | |
| Marital Status | **1.31*** | | **1.30*** | | **1.28*** | | **1.33*** | **1.29*** | |
| Education | 0.83 | | 0.85 | | 0.86 | | 0.84 | 0.86 | |
| Sleep | **0.78*** | | **0.76*** | | **0.78*** | | **0.78*** | **0.78*** | |
| Self-Rated Health | **1.43*** | | **1.49*** | | **1.46*** | | **1.42*** | **1.44*** | |
| Activities of Daily Living | 1.01 | | 1.01 | | 1.01 | | 1.01 | 1.01 | |
|  |  | |  | |  | |  |  | |
| *Mediator/Moderator* |  | |  | |  | |  |  | |
| Social Support | 0.89 | |  | |  | | 0.81 |  | |
| Neighborhood Collective Efficacy |  | | 0.84 | |  | |  |  | |
| Number of Close Friends |  | |  | | 0.95 | |  | 0.98 | |
|  |  | |  | |  | |  |  | |
| *Interaction Term* |  | |  | |  | |  |  | |
| MD X Social Support |  | |  | |  | | 1.19 |  | |
| MD X Friends |  | |  | |  | |  | 0.86 | |
|  |  | |  | |  | |  |  | |
| Model Coefficients | χ2(10) = 658.65*** | | χ2(10) = 658.85*** | | χ2(10) = 668.46*** | | χ2(11) = 659.46*** | χ2(11) = 670.25*** | |
| Nagelkerke R^2^ | 0.46 | | 0.46 | | 0.46 | | 0.46 | 0.46 | |
| * p<0.05, ** p<0.01, *** p<0.001 MD - Material Deprivation, Friends - Number of Close Friends | | | | | | | | | |
